# DFOS Technology in Geoengineering Monitoring in the Past 35 Years: A Bibliometric Analysis

**DOI:** 10.3390/s24155051

**Published:** 2024-08-04

**Authors:** Jia Wang, Ankit Garg, Neelima Satyam, Askar Zhussupbekov, Svetlana Sushkova

**Affiliations:** 1 School of Earth Sciences and Engineering, Nanjing University, Nanjing 210023, China; jiawang@smail.nju.edu.cn; 2 Department of Civil and Intelligent Construction Engineering, Shantou University, Shantou 515063, China; 3 Department of Civil Engineering, Indian Institute of Technology Indore, Indore 453552, Madhya Pradesh, India; neelima.satyam@iiti.ac.in; 4 Department of Civil Engineering, L.N. Gumilyov Eurasian National University, Astana 010008, Kazakhstan; astana-geostroi@mail.ru; 5 Academy of Biology and Biotechnology, Southern Federal University, Rostov-on-Don 344090, Russia; snsushkova@sfedu.ru

**Keywords:** geoengineering, DFOS, Web of Science, bibliometric analysis, VOSviewer

## Abstract

DFOS (distributed fiber-optic sensing) technology has shown the potential to increase the accuracy of measurement after years of development and experimenting in geoengineering monitoring. To better understand the development of DFOS technology and its contribution to geoengineering, an objective and data-driven review of the development process of DFOS technology in construction was completed. The review was accomplished by using text mining methods on the Web of Science, covering a wide range of relevant data, including 3970 articles from 1989 to 2023. The results indicate that DFOS technology research demonstrates the typical characteristics of multi-author, multi-country, and multi-institution collaborations, spanning various research fields. Over the past 35 years, the number of published articles has exhibited exponential growth, with China making significant contributions and leading in terms of its total publication growth rate, which has been higher than that of the United States since 2016. In the analysis of author keywords, emerging technologies, such as machine learning and distributed acoustic sensing, have garnered attention. The findings contribute to a comprehensive understanding of the development, impact, and future trends of DFOS technology in geotechnical engineering, offering valuable insights for researchers, scholars, and students in the field and inspiring new approaches for research methods in this domain.

## 1. Introduction

Geoengineering refers to geological and geotechnical engineering, which is a discipline that uses engineering and geology information to solve practical engineering problems, such as those concerning landslides, tunnel stability, pipeline leakages, and land subsidence [1,2,3]. These problems are expected to be effectively prevented or solved by carrying out risk assessments and management through data analysis [4]. To obtain effective field data, reliable engineering monitoring methods are needed, especially for large geotechnical construction processes [5].

**Figure 1:**
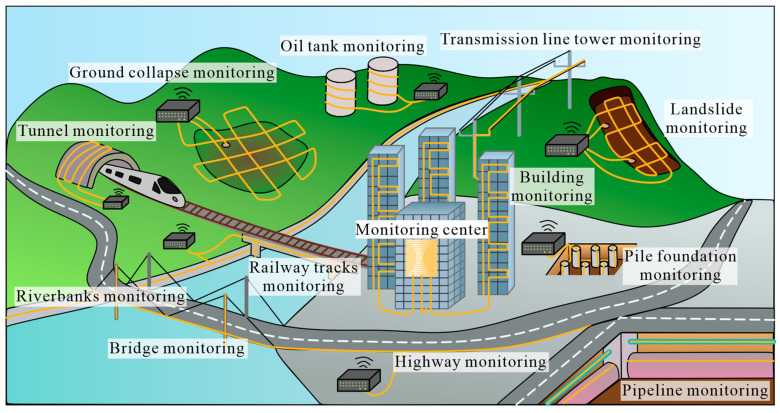
Schematic illustration of the application of DFOS technology in geoengineering monitoring.

Distributed fiber-optic sensing (DFOS) technology is a new type of sensing technology that was rapidly developed in the 1980s [6]. In recent years, due to the requirement and advantages of deformation, temperature, and pressure monitoring, DFOS technology has made a lot of progress in the field of the monitoring of structures [7,8,9,10,11]. An illustration of the application of DFOS technology in geoengineering monitoring is shown in Figure 1.

The application of DFOS technology in geoengineering monitoring has gradually become a hot topic in recent decades. For example, the Belt and Road Initiative (BRI), a development strategy adopted by the Chinese government involving infrastructure development and investments in 155 countries and international organizations in Europe, Asia, the Middle East, Latin America, and Africa, was announced by Xi Jinping in 2013. During the implementation of this strategy, DFOS technology plays an important role in monitoring applications, such as bridges, ports, railways, and other infrastructure. Therefore, it is important to understand the current development situation of DFOS technology in this research field. 

One effective way is to conduct a quantitative analysis, including statistical assessments, of the published papers within this field through a bibliometric analysis. This article conducts a comprehensive quantitative analysis of the literature on DFOS technology in geoengineering monitoring over the past 35 years. This analysis provides insights into the impacts of countries, research institutions, publication sources, and authors in this field. Additionally, it examines research trends based on keywords. Based on these analyses, this article offers pertinent suggestions related to this study.

## 2. A Brief History of DFOS

**Figure 2:**
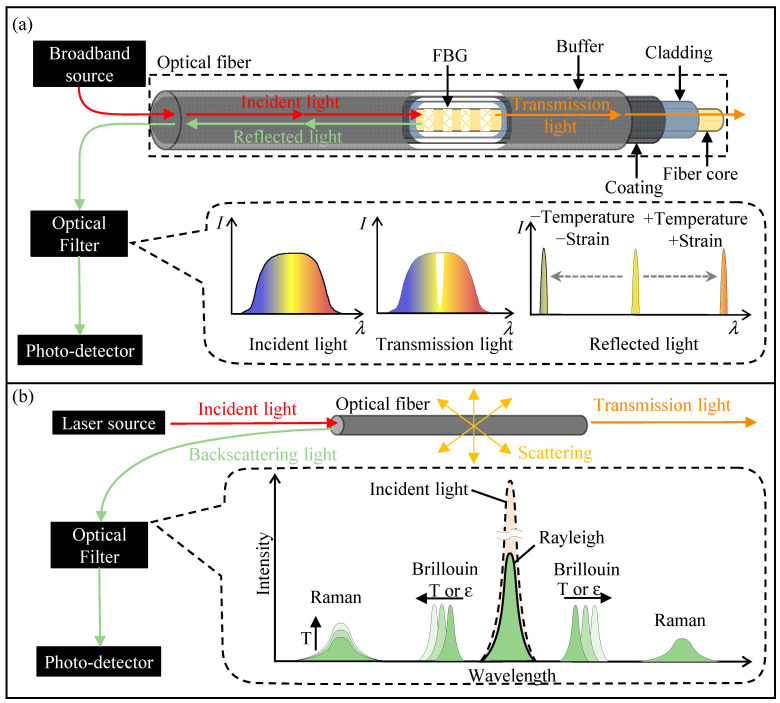
Principles of DFOS technologies: (**a**) Quasi-distributed technology; (**b**) Fully distributed technology.

Optical fibers, as illustrated in Figure 2, serve as the “sensing nerves” of the ground in DFOS technology. Their basic structure typically consists of a fiber core and cladding. The core, with a diameter ranging from 5 to 75 μm, is primarily composed of silica, with a small amount of other materials doped to enhance its refractive index. The cladding is a material layer closely surrounding the core, with a diameter usually ranging from 100 to 400 μm, and its optical refractive index is slightly lower than that of the core material. The core facilitates the transmission of optical signals, while the cladding encloses light within the core, protecting it and reinforcing the mechanical strength of the fiber. 

DFOS technologies can be divided into two types: quasi-distributed and fully distributed. Quasi-distributed technology, also known as serial fiber-optic sensing technology, connects multiple sensors through a single optical fiber. It uses techniques such as time division multiplexing, frequency division multiplexing, and wavelength division multiplexing to form a multi-point fiber optic sensing system. The most common technique is fiber Bragg grating (FBG) sensing technology, the sensing principle of which is shown in Figure 2a. When a broadband source passes through an optical fiber containing FBG, the light satisfying the Bragg diffraction condition is reflected at the FBG, and the reflection spectrum shows a peak at the FBG center wavelength. When optical fibers are exposed to external factors such as strain or temperature, the center wavelength of the reflection spectrum shifts, and by measuring the shift in the center wavelength, the corresponding changes in strain and temperature can be determined [12,13]. The working principle of fully distributed fiber-optic sensing technology is mainly based on light scattering. As shown in Figure 2b, backscattering occurs when light is transmitted in the fiber. According to the scattering mechanism, the scattered light in the fiber can be divided into Rayleigh-scattered light, Brillouin-scattered light, and Raman-scattered light. Rayleigh scattering is elastic scattering, and the frequency of the scattered light does not shift. Since the ratio of Rayleigh-scattered light intensity to the transmitted light power is a constant, it is mainly used to measure the location and extent of fiber breakpoints and damage [14,15]. Brillouin scattering and Raman scattering are both inelastic scattering, and the frequency of the scattered light shifts during the scattering process [16,17]. Brillouin-scattered light shifts in frequency under external forces or temperature changes. Based on the linear relationship between Brillouin frequency shifts and temperature/strain, the temperature and strain along the fiber can be determined. The intensity of Raman-scattered light is only related to temperature, so it is mainly used for temperature measurements along fibers.

**Figure 3:**
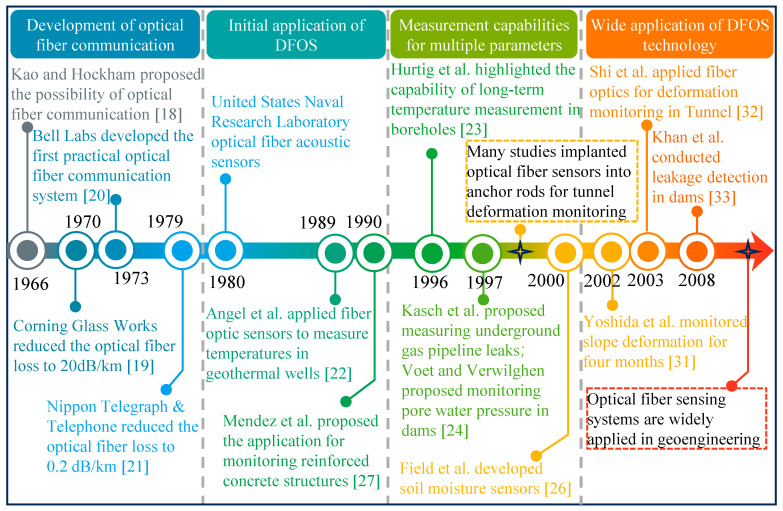
Development history of DFOS technology in geoengineering monitoring.

As shown in Figure 3, the concept and technology of optical fiber communication were first introduced by Charles K. Kao and George A. Hockham in 1966. They identified that the attenuation in fibers was due to impurities that could be removed. They suggested that high-purity silica could reduce optical loss to below 20 dB/km, laying the foundation for optical fiber communication [18]. In 1970, Robert D. Maurer, Donald Keck, Peter C. Schultz, and Frank Zimar at Corning Glass Works in the United States achieved an optical fiber with a loss of 17 dB/km by silica glass with titanium, which garnered significant attention [19]. By 1973, Bell Labs had developed the first practical fiber-optic communication system, marking the transition of fiber-optic communication from theoretical research to practical application [20]. Over the next decade, researchers focused on improving the efficiency of optical signals, and by 1979, Nippon Telegraph and Telephone had reduced optical fiber loss to 0.2 dB/km, leading to significant growth in optical fiber communication. This revolutionized communication networks and spurred the exploration of optical fiber applications in various fields [21]. The development of optical fiber sensors began in the 1980s, with the US Naval Research Laboratory creating acoustic sensors to monitor underwater submarine sounds. In 1989, optical fiber sensors were first used for on-site temperature measurements in geothermal wells, laying a foundation for their integration into geotechnical engineering monitoring [22]. In the 1990s, scholars focused on the measurement capabilities of optical fiber sensors for different parameters. During this period, optical fiber sensors successfully monitored temperature, strain, moisture content, and pore water pressure in laboratory experiments [23,24,25,26,27]. Simultaneously, optical fiber sensors were applied to deformation monitoring in practical engineering [28,29,30]. In the 21st century, DFOS technology gained widespread attention. The application of optical fiber sensors expanded to safety monitoring in slopes, tunnels, dams, and other engineering projects [31,32,33,34,35,36,37]. Integrated with advanced data acquisition systems, optical fiber monitoring systems recorded real-time data and transmitted them remotely, proving to be significant in evaluating the stability of underground structures and providing warnings of geological disasters.

Due to its suitability for extensive and long-distance distributed monitoring in geological and geotechnical engineering, DFOS technology has become a research focus and significant scientific endeavor in some developed countries internationally, including Japan, Switzerland, Canada, United States, South Korea, Italy, and France. Early research efforts were primarily concentrated on implanting FBG sensors into fiber-reinforced polymer (FRP) anchors and tendons, applying them to monitor deformations in tunnels or roadways surrounding rock [28]. At present, a series of DFOS technologies have been successfully applied to geoengineering monitoring [17,38]. This includes FBG, ultra-weak FBG (UWFBG), Brillouin optical time/frequency-domain reflectometers and analyses based on the Brillouin scattering principle (BOTDR, BOTDA, and BOFDA), Raman optical time/frequency-domain reflection technology based on the Raman scattering principle (ROTDR and ROFDR), and optical time-domain reflectometer/phase-sensitive optical time-domain reflectometer technology based on the Rayleigh scattering principle (OTDR, Φ-OTDR).

Table 1 shows DFOS technology and its features in geoengineering monitoring. Moreover, DFOS technology mainly realizes real-time monitoring by sensing changes in temperature, strain, or vibration. Based on these monitoring parameters, DFOS can be categorized into distributed temperature sensing technology (DTS), distributed strain sensing technology (DSS), and distributed acoustic technology (DAS) [32,39,40,41]. Currently, through continuous exploration by scholars, DFOS technology based on DSS, DTS, and DAS can monitor key parameters such as strain, temperature, moisture content, and stress in various research areas of geoengineering monitoring, as shown in Table 2. 

## 3. Database Selection and Data Mining Process

In this review, the WoS (Web of Science) database was used for data mining. The WoS is the most complete and reliable database for bibliometric analyses, containing more than 12,000 high-impact and high-quality academic journals. The core collection of the WoS was selected in this paper. Furthermore, using the advanced search function of this website, field identifiers, Boolean operators, parentheses, and search results can be used to perform searches. In the processes of retrieval, different topics of DFOS technologies and geoengineering were retrieved with no further restrictions on language or literature type. The following are the concrete processes used.

### 3.1. Step One: Determine the Research Topics

In this step, the main research topics of DFOS technology and geoengineering monitoring were selected and retrieved, and articles of unrelated subject categories were removed. In this process, “TS” means research topics, and “SU” means research areas. The following is the retrieval process: 

TS = (fiber-optic OR optical-fiber OR FBG OR BOTDA OR BOTDR OR BOFDA OR ROFDR OR ROTDR OR OFDR OR OTDR OR “distributed strain sensing” OR “distributed temperature sensing” OR “distributed vibration sensing” OR “distributed acoustic sensing”) AND TS = (geotechnical OR geological OR geologic OR geothermal OR geophysics OR strata OR soil OR rock OR landslide OR subsurface OR pile OR slope-stability OR ground-movement OR debris-flow OR slope-excavation OR underground OR embankment OR slope-failure OR ground-fissure OR geothermal OR land-subsidence OR borehole) NOT SU = (Astronomy Astrophysics OR Archaeology OR Architecture OR Agriculture OR Anatomy Morphology OR Art OR Business Economics OR Biochemistry Molecular Biology OR Biophysics OR Crystallography OR Dentistry Oral Surgery Medicine OR Education Educational Research OR Food Science Technology OR Gastroenterology Hepatology OR Imaging Science Photographic Technology OR Integrative complementary medicine OR Life Sciences Biomedicine Other Topics OR Legal Medicine OR Microscopy OR Microbiology OR Mathematics OR Neurosciences Neurology OR Ophthalmology OR Operations Research Management Science OR Public Environmental Occupational Health OR Plant OR Robotics OR Polymer Science OR Public Administration OR Paleontology OR Radiology Nuclear Medicine Medical Imaging OR Research Experimental Medicine OR Rehabilitation OR Social Issues OR Surgery OR Toxicology OR Zoology).

### 3.2. Step Two: Retrieve Information on Other Topics in Geoengineering Monitoring

In addition to geological hazard monitoring, DFOS technology is also widely used in other engineering monitoring fields, such as bridge foundations, tunnels, and highways. Therefore, information pertaining to its other applications in monitoring can be retrieved by excluding specific terms and irrelevant subject categories. The following is the retrieval process:

TS = (fiber-optic OR optical-fiber OR FBG OR BOTDA OR BOTDR OR BOFDA OR ROFDR OR ROTDR OR OFDR OR OTDR OR “fiber Bragg grating” OR “distributed strain sensing” OR “distributed temperature sensing” OR “distributed vibration sensing” OR “distributed acoustic sensing”) AND TS = (tunnel OR ground collapse OR oil tank OR pile OR foundation OR bridge OR riverbank OR highway OR railway OR pipeline) NOT TS = (side-tunnel OR tunnel-coupled OR tunnel-diode OR tunnel-lens OR scanning tunneling OR machine-tunnel OR soliton-tunneling OR pure-tunneling OR micro-tunneling OR tunnel-effect OR MRI-tunnel OR wind-tunnel nanostructure OR communication OR protection OR discrimination OR vibrations OR error OR pulse OR probe OR inorganic OR organic OR composite laminates) NOT SU = (Automation control system OR Agriculture OR Audiology Speech language pathology OR Astronomy Astrophysics OR Anesthesiology OR Biotechnology Business OR Biochemistry Molecular Biology OR Crystallography OR Cardiovascular system cardiology OR Chemistry OR Cell biology OR Dentistry Oral Surgery Medicine OR Education educational OR Experimental medicine OR Food OR Food Science Technology OR Fisheries OR Government law OR Gastroenterology Hepatology OR History OR Imaging Science Photographic Technology OR Microscopy OR Mathematics OR Medical Laboratory technology OR Nuclear Science Technology OR Neurosciences Neurology OR Nutrition dietetics OR Ophthalmology OR Operations research management science OR Physics OR Public Administration OR Polymer science OR Public Environmental Health OR Radiology Nuclear Medicine Medical Imaging OR Rehabilitation OR Research Experimental Medicine OR Robotics OR Surgery OR Spectroscopy OR Telecommunications OR Thermodynamics OR Urban).

### 3.3. Step Three: Combine Retrieval Forms

In this step, the above retrieval results were combined into one, and the final retrieval results represented their intersection. The following is the retrieval process:

(#1 OR #2) NOT (TS = (Pile-up OR Mile-rock) OR SU = (Agricultural OR Agriculture OR Agronomy OR Anesthesiology OR Business Economics OR Biophysics OR Biochemistry OR Biosystems OR Cardiovascular System Cardiology OR Crystallography OR Cell OR Chemistry OR Chemical OR Crops OR Dermatology OR Electrochemistry OR Entomology OR Endocrinology Metabolism OR Education OR Electrical OR Films OR Freshwater biology OR Genetics OR Gerontology OR Heart OR Health OR Horticulture OR Hematology OR Immunology OR Language OR Legume OR Library OR Music OR Material OR Microbiology OR Mathematical OR Medicine OR Medical OR Nuclear OR Nutrition Dietetics OR Oncology OR Otorhinolaryngology OR Photographic Technology OR Pharmacology Pharmacy OR Psychiatry OR Plant OR Power delivery OR Production operation OR Rheumatologist OR Respiratory OR Semiconductor OR Society OR Social OR Signal OR Urology Nephrology)) AND PY = (1989–2023).

In this process, “#1” and “#2” represented the first two results, respectively, and some terms unrelated to geoengineering monitoring were eventually excluded. Further, the period was customized from 1989 to 2023. The earlier literature primarily focused on the study of the optoelectronic characteristics of fiber-optic components [66], experiments on fiber-optic communication capabilities [67,68], and the design and exploration of applications for environmental variable monitoring [69]. However, comprehensive laboratory experiments or field applications in geoengineering monitoring were not conducted or implemented during this earlier period.

Through the above steps, 3970 related articles were obtained. These articles can be classified and counted by publication years, subject categories, authors, countries, and research institutes on the WoS, and citation reports can be generated automatically. Furthermore, the information of these articles can be exported as plain text files, which can be analyzed by the R language platform (version 4.3.0), the bibliometrix R-package (version 4.0.0), the biblioshiny web app, and VOSviewer software (version 1.6.20). After an analysis, the links between different countries, research institutes, authors, and keywords can be displayed in corresponding network maps. These software programs were chosen due to their suitability and frequent use in works similar to the current research [70,71]. The results of the above analysis are described in detail in the next section.

## 4. Results

### 4.1. Overview

**Figure 4:**
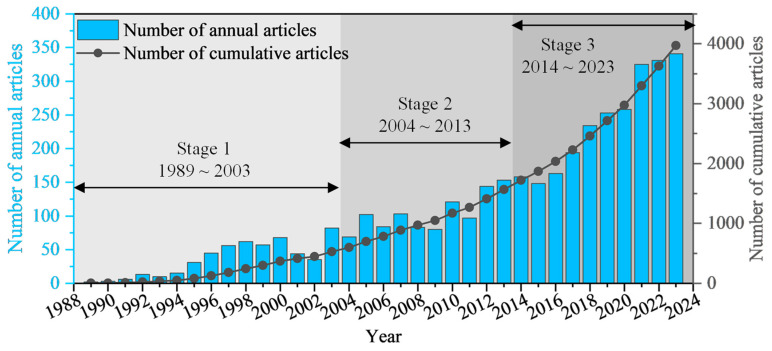
Changes in the number of published articles and countries from 1989 to 2023.

Figure 4 shows the evolution of the main variables in this field on a global scale from 1989 to 2023, which is useful to understand the overall characteristics. The first related article was “Field applications of fiber-optic sensors. Part I: Temperature measurements in a geothermal well” [22], published in the journal of Applied Spectroscopy in 1989. In this article, the author uses fiber-optic sensors for the first time to measure the temperature of in situ geothermal wells, which is of great significance for the application of DFOS in geoengineering monitoring. 

A further analysis reveals that the literature in this field has increased at a relatively low rate in the years after 1989. By the end of 2003, the cumulative number of published articles in the previous 15 years had reached 529, with an average of 35 published annually. There was an increase in the number of publications from 2004 to 2013, with 104 annual published articles on average. The outbreak period is 2014~2023. In this period, with the rapid development of research, the cumulative number of articles increased significantly, reaching 2405 and accounting for 60.58% of the total. The average annual number of articles reached 241. Therefore, it can be concluded that the research development in this field can be divided into three stages: germination, slow development, and rapid development, which indicates that this field has attracted more and more attention from researchers.

### 4.2. Research Areas and Technology Applications

**Figure 5:**
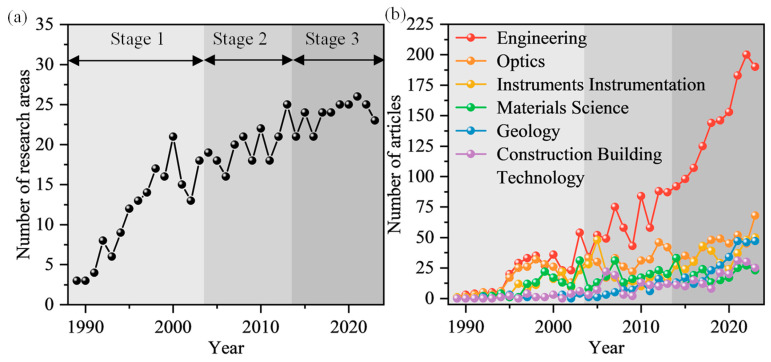
Trend of research areas for DFOS-based geoengineering monitoring: (**a**) number of research areas by year; and (**b**) number of articles of the top 6 most productive WoS research areas.

In the WoS database, the retrieved articles are divided into 31 research areas. Figure 5 shows the evolution of the six main subject categories. Note that an article may be simultaneously included in more than one category. During the period studied, 59.67% of published articles were in Engineering, 24.74% were in Optics, 17.28% were in Instruments & Instrumentation, 12.92% were in Materials Science, 9.29% were in Geology, and 7.73% were in Construction Building Technology. Since the beginning of the analyzed period, there have been numerous articles published in the areas of Engineering, Optics, and Instruments & Instrumentation. Since 1995, the number of publications in Engineering and Optics has started to increase faster than in other fields. Since 2000, Engineering has become the leading subject category in this field, which indicates that the related research is being principally conducted from an engineering perspective. After 2003, the number of publications in Optics, Instruments & Instrumentation, and Materials Science has remained relatively stable. The number of publications related to Geology and Construction Building Technology initially increased slowly, but it started to increase more rapidly from 2018. This trend suggests a shift in focus towards engineering solutions and technological advancements in geoengineering. As the field continues to evolve, interdisciplinary collaborations between engineering, optics, materials science, and geology are likely to drive further innovations in research and practical implementations.

In the past 35 years, DFOS technology has been applied to the monitoring of pipelines, slopes, tunnels, foundations, and other engineering projects [72,73]. Meanwhile, relevant model tests and feasibility studies have also been continuously carried out [74,75,76]. With the continuous advancement of research, relevant monitoring guidelines and standards are gradually being formed [77].

**Figure 6:**
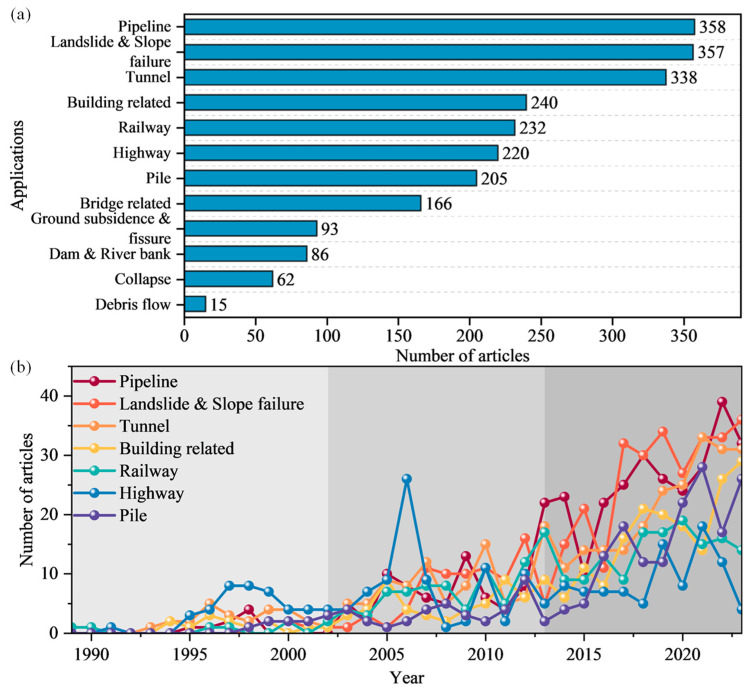
Statistical information on DFOS technology monitoring in geoengineering by application scenarios: (**a**) Total scientific production; and (**b**) annual production of top 7 applications.

Figure 6a shows the number of publications related to the application of DFOS technology in various monitoring fields. It is evident that DFOS technology has made significant advancements in monitoring geological hazards, such as landslides, debris flows, ground subsidence, and ground fissures, as well as in engineering, such as foundations, pipelines, tunnels, and highways [16,49,61,78,79]. Figure 6b illustrates the changes in the number of publications over time for the top seven applications. After 1995, the number of publications in various monitoring fields has grown, corresponding to the increase in related research publications, as shown in Figure 5b. Among the various monitoring fields, pipelines, slopes, tunnels, and foundation monitoring are the main application areas for fiber-optic monitoring, with a consistently high number of publications and varying degrees of growth in recent years. The number of publications on pile monitoring has been steadily increasing in Stage 1 and Stage 2, with a rapid growth after 2013, indicating the potential of DFOS technology in pile monitoring. In the fields of highway and railway monitoring, the number of publications on DFOS technology has shown a decreasing trend in recent years. The number of publications on highway monitoring peaked in 2006, partly due to the continuous development of DFOS technology, leading scholars to explore and apply it in different fields, and partly due to the numerous international conferences held that year, such as the Second International Conference on Structural Health Monitoring of Intelligent Infrastructure, Smart Structures and Materials 2006 Conference, and Conference on Optical Sensing II. These conferences resulted in a significant number of publications related to highway roadbeds, buried pipelines, and highway tunnels, leading to a surge in the number of publications that year. Overall, DFOS technology has demonstrated substantial growth and versatility across various engineering and geological applications. Its continued development and increasing adoption underscore its critical role in modern infrastructure monitoring and disaster prevention.

### 4.3. Technology Categories

According to the type of DFOS technology, the retrieved articles can be divided into several groups. The four major technology categories are shown in Table 3. Of the total 1184 articles, 72.6% were related to FBG, 9.4% were related to OTDR, 9.2% were related to BOTDR, and 8.9% were related to BOTDA. FBG-related articles are leading in terms of number, citations, and H-index. The first article related to FBG on the WoS was published in 1997. In the early 1990s, the manufacturing of FBG sensors became mature and therefore significantly promoted the wide application of this technology to different engineering disciplines [80]. Due to their relatively low cost, ease of multiplexing, and capability of measuring strain and temperature, FBG-related research and development in geoengineering monitoring have been continuously carried out and occupied a dominant position over the past 35 years. Articles related to BOTDR technology have the highest average citation rate of 19.44, but the total number of published articles is moderate. Due to cost constraints, this technology is not the most commonly used in geoengineering monitoring, but it has a high level of recognition. Articles related to BOTDA were first published in 2008. Incidentally, BOTDA technology was officially introduced in the late 1980s. However, it was not until many years later that it was applied to geoengineering and only then were relevant articles included in the WoS. This lag is also evident in other DFOS technologies. For example, the first FBG fiber was made by Hill in 1978 [81], but it was 20 years later that FBG technology was applied to monitor rockbolts and ground anchors. The reason for this phenomenon is that the great importance of automatic and geotechnical instrumentation has only been recognized in recent years. In addition, geoengineering is a relatively conservative discipline, and the acceptance of cutting-edge technologies such as DFOS was not very high in early years. Unlike the previously mentioned technologies, OTDR technology is primarily used for distributed vibration sensing or distributed acoustic sensing. It can monitor breakpoints and light intensity but cannot directly measure temperature and strain changes. Additionally, this technology has a high level of sensitivity, which results in a large amount of noisy data during monitoring. Consequently, compared to FBG technology, OTDR was not widely used until recently. 

**Figure 7:**
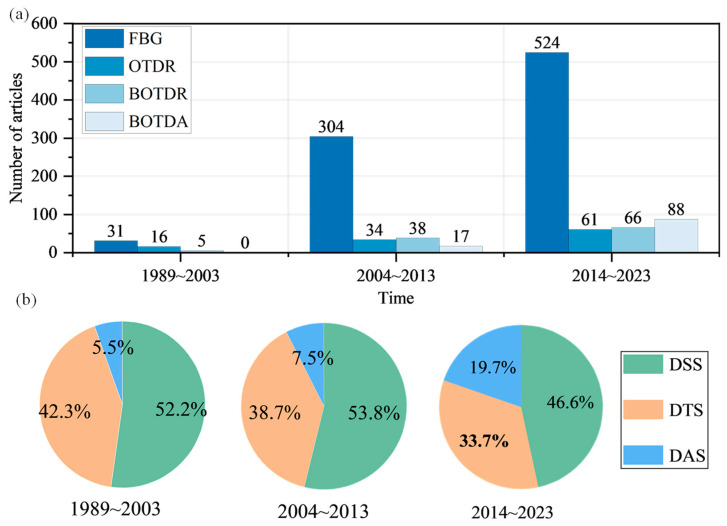
(**a**) Number of articles on major technology categories in different periods; (**b**) The percentage of different sensing parameters in different periods.

Figure 7a shows changes in the number of articles related to the above-mentioned technology categories applied to geoengineering monitoring over the past 35 years. In Stage 1 (1989~2003), only FBG, BOTDR, and OTDR technologies were applied to geoengineering monitoring. In Stage 2 (2004~2013), BOTDA was first applied to geoengineering monitoring in 2008. The total number of relevant research articles related to these four technologies reached 393, of which 304 were related to FBG. This shows that in this period, relevant research began to be conducted in specific directions, among which FBG-related research accounted for the largest proportion. In Stage 3 (2014~2023), the number of articles published on geoengineering monitoring using four types of fiber-optic monitoring technologies reached 739, of which 524 were related to FBG, 66 were related to BOTDR, 88 were related to BOTDA, and 61 were related to OTDR. In this period, relevant research was developed, especially regarding FBG. Figure 7b shows the changes in the proportion of research and strain, early applications of DAS were not widespread. This is mainly because OTDR mainly detects the occurrence of accidents by monitoring breakpoints and light intensity and cannot quantitatively measure physical parameters that geoengineering practitioners care about. Additionally, due to its high sensitivity, OTDR generates a large amount of noisy data, and the processing of these noisy data was one of the challenges faced by early scholars. Over time, research on DAS has gradually increased, and its proportion has also steadily risen. In recent years, advancements in computer technology have made the bulk processing of large amounts of data possible, leading to increased research and application of this refined, high-sensitivity detection method.

### 4.4. Countries and International Cooperation

**Figure 8:**
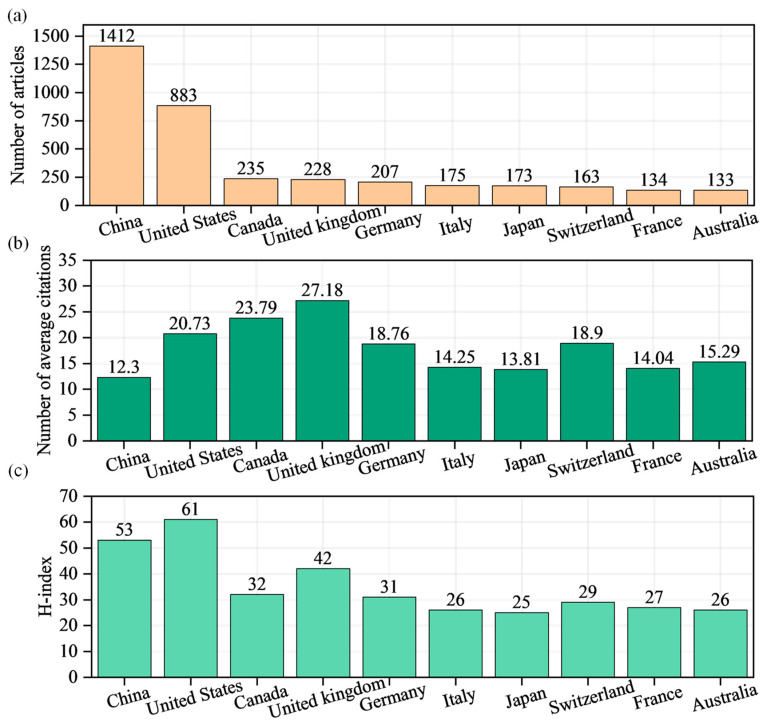
The top 10 most published countries: (**a**) total number of publications; (**b**) average number of citations; (**c**) H-index of publications.

The cumulative number of publications is an important indicator for measuring the scientific research strength of a country or region in a certain research field. The H-index is currently widely accepted as one of the benchmarks for measuring scientific performance, and a higher H-index indicates a scientist has a greater influence [71,86,87]. In order to achieve the goals of evaluation and comparison, we calculated the H-index for publications from each country and displayed the results in Figure 8c.

Figure 8a shows the top 10 most productive countries in terms of the publication of articles in this field. China led the group, followed by the United States, Canada, United Kingdom, Germany, Italy, Japan, Switzerland, France, and Australia. Regarding the average citation count shown in Figure 8b, the United Kingdom (27.18), Canada (23.79), and United States (20.73) exhibit relatively high levels, while Japan (13.81) and China (12.3) have comparatively lower levels. Combining the corresponding H-index, as depicted in Figure 8c, we observe that, although China has a high number of published articles and a high H-index, the average number of citations is significantly lower than the other nine countries. This indicates that overall, China’s international influence in this field is relatively high, second only to the United States. However, due to varying publication quality, Chinese scholars in this field have not received consistent recognition from scholars in other countries, resulting in a lower average citation count for Chinese articles. The United States, with high numbers of publications and citations, demonstrates higher publication quality, earning recognition from industry peers and thus maintaining the highest H-index. Compared to China and the United States, European countries generally have lower publication numbers, with the United Kingdom showing advantages in both publication quantity and impact compared to other European countries. Similar to China, Italy and Japan have relatively high publication numbers but their international influence does not match their publication quantity, indicating that the overall quality of publications still needs to be improved.

**Figure 9:**
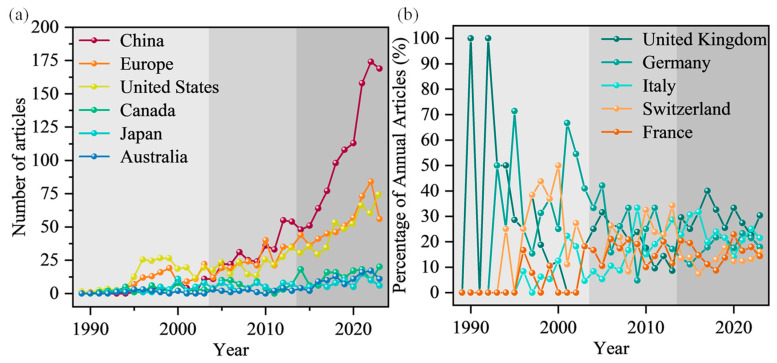
Statistical information on DFOS technology monitoring in geoengineering by country: (**a**) annual scientific production of the top 10 countries and (**b**) annual proportions of the countries with a cumulative number of documents over 250.

Figure 9a depicts the changes in publication numbers over time for the top 10 most productive countries. For clarity in observing the overall trends, five European countries including the United Kingdom, Germany, Italy, Switzerland, and France are grouped as “Europe” in Figure 9a. Figure 9b illustrates the percentage of publications from the five European countries relative to the total annual publications. It can be observed that China has shown the most significant growth, with an average of only two articles published per year during 1989–2003, increasing to an average of 106 articles published per year during 2014–2023. The United States and Europe have maintained comparable publication levels, initially leading in this field. Canada, Japan, and Australia have consistently published articles each year, although in smaller and relatively stable numbers. China entered research in this domain in 1994, achieving publication levels comparable to Europe and the United States by 2004–2005, surpassing them in cumulative publications by 2016, and currently maintaining a leading position in this field. Figure 7b indicates that among European countries, the United Kingdom and Germany have conducted extensive research in this field. In recent years, Italy, France, and Switzerland have also increased their publication numbers in this domain, with proportions generally comparable to the United Kingdom and Germany.

**Figure 10:**
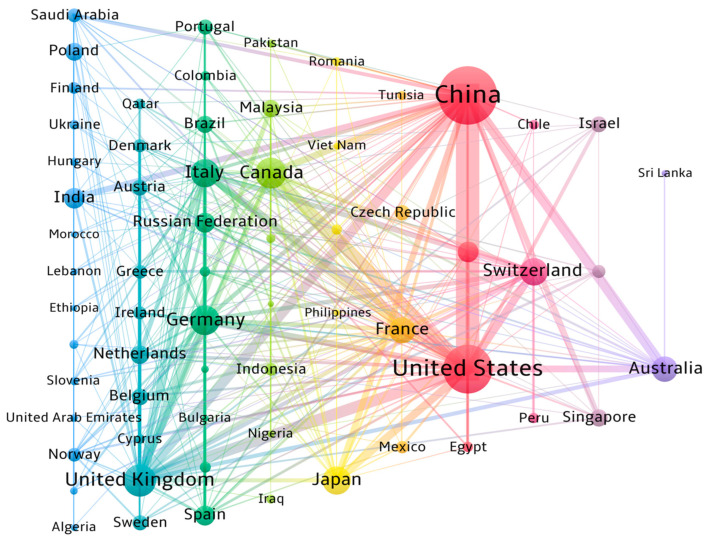
Map showing research collaborations between countries. Different colors represent different clusters; the size of a circle represents the number of publications in a country ; and the width of the connecting lines represents the number of collaborative publications between two countries .

In addition to the number of published articles and citation counts, the frequency of international collaborations is also a crucial indicator reflecting a country’s research prowess. We identified 65 countries with more than three published articles in this analysis. The collaboration patterns between countries are shown in Figure 10. The thickness of the connecting lines represents the frequency of collaboration between countries, and the top five countries with the most collaborations are, in descending order, the United States (371), China (312), the United Kingdom (266), Canada (126), and France (122). The top five countries with the highest number of collaborative partners are the United Kingdom (64), China (57), the United States (40), France (35), and Canada (28). The results indicate that, although the United States and France have a similar number of collaborating countries, the frequency of collaboration and the volume of published articles in the United States are more than double those of France, further confirming the United States’s leadership in research within this field. This is consistent with the article published by Chawla in 2018 [88]. The article states that the United States is the largest collaborator with 202 connections in different countries followed by United Kingdom. One possible reason for strong collaboration could be the highest number of international graduates [89]. The migration of some or many international graduates to their home country or other countries might have also helped in building partnerships with the United States and United Kingdom.

According to specific collaborating countries, countries that engage in frequent collaborations generally wield significant influence within their respective fields, correlating closely with the number of published articles. The countries with the highest collaboration frequencies include the United States and China (93), the United States and Canada (40), the United States and the United Kingdom (31), China and Australia (30), and China and the United Kingdom (28). These collaborations underscore the pivotal role of international partnerships in advancing the application of fiber optics in geotechnical engineering. For instance, cooperation between the United States and China has contributed significantly to the application of DAS for seismic and geothermal monitoring [90,91,92]. Collaboration between China and Australia has made substantial progress in deformation monitoring of structures such as piles and bridges [93,94]. These collaborations often involve multidisciplinary teams and leverage each country’s unique expertise and resources to provide innovative solutions to complex geotechnical challenges.

### 4.5. Research Institutions and Cooperations between Them

**Figure 11:**
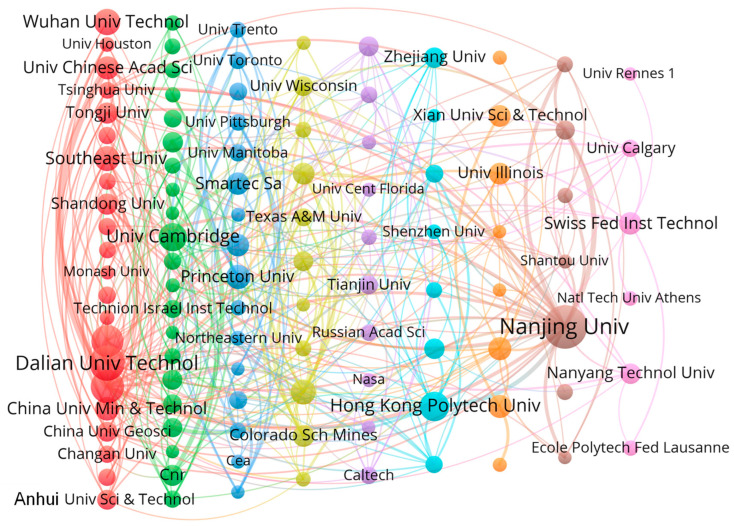
Map showing research collaborations between institutions. Different colors represent different clusters; the size of a circle represents the number of publications in an institution; and the width of the connecting lines represents the number of collaborative publications between two institutions.

Based on the co-authorship analysis type, ignoring articles with more than 25 authors and institutions with less than 10 published documents, we extracted information on cooperative institutions engaged in DFOS monitoring in geoengineering, as shown in Figure 11. The results indicate that there are 109 research institutions engaged in this field. The top 20 institutions with the highest number of publications and their countries, as well as the number of citations, are shown in Table 4.

As shown in Table 4, the research institutions in this field have significant differences in terms of publication quantity and impact across various organizations. The research institutions are concentrated in China (9), followed by the United States (4), Germany (2), the United Kingdom (2), and Switzerland, France, and Italy (1 each). The Nanjing University and Dalian University of Technology, located in China, lead in the number of publications with accumulated counts of 169 and 107, respectively. Following these is the United States Department of Energy with a publication count of 104. Similar to the previous analysis based on countries, although the Nanjing University and Dalian University of Technology match or exceed research institutions in developed countries such as the United States and United Kingdom in terms of publication quantity, their articles have a significantly lower overall citation rate compared to other institutions. This also underscores the continued significant impact and leadership of the United States in this field, maintaining a competitive advantage over various countries. 

In terms of cooperation, Chinese institutions have a clear advantage in the number of collaborations with domestic institutions, and this cooperation is closely related to geographical location. For example, the Chinese Academy of Sciences has collaborated 35 times with the University of Chinese Academy of Sciences, the Dalian University of Technology has collaborated 22 times with the Harbin Institute of Technology, and Nanjing University has collaborated 19 times with Suzhou Nanzee Sensing Technology Co., Ltd (Suzhou, China). This pattern is largely due to China still being a developing country, and international collaborations and opportunities have significant costs. Despite a large number of international graduates facilitating closer international collaborations, collaborations between geographically proximate domestic institutions are easier to establish and thus strengthen. In contrast, for other developed countries, the costs of international and domestic collaborations are relatively similar, with the primary goal being resource integration. Therefore, collaborations between institutions from different countries are often closer. For instance, the University of California, Berkeley in the United States has collaborated 10 times with the University of Cambridge in the United Kingdom. Additionally, Lawrence Berkeley National Laboratory in the United States has collaborated 14 times with Rice University, also in the United States, and nine times with Curtin University in Australia.

### 4.6. The Impacts of Authors

Table 5 shows the top 20 core authors ranked by H-index in this field. Given the relatively small scope of this field, the H-index is based solely on an author’s publications within it. Consequently, a lower H-index may be attributed to scholars engaging in research across multiple fields. In comparison to an author’s overall H-index across all fields, the H-index within this specific field tends to be smaller. 

**Figure 12:**
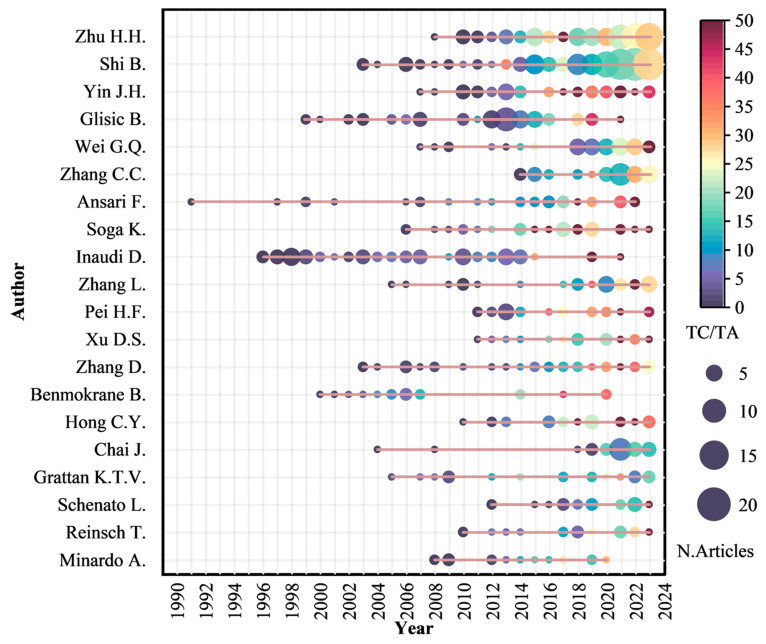
Top 20 authors’ productivity over time, ranked by H-index. The color of the circles present the average number of citations in that year; the size of the circles present the number of publications in that year; the red line presents the duration of the author’s research in this field; TC/TA means the average number of citations per year; N.Articles means the number of published articles.

Table 5 reveals the top six authors with H-index exceeding 15: Zhu H.H. (28), Shi B. (26), Yin J.H. (19), Glisic B. (18), Wei G.Q. (16), and Zhang C.C. (16). Except for Glisic B., all of these scholars are from China. In terms of article numbers, Shi B. (120) and Zhu H.H. (84) from Nanjing University in China secured the top two positions, establishing them as prolific authors. Figure 12 shows the authors’ production over time, it can be observed that the research output of these two authors has consistently continued over the years. This indicates that Nanjing University has been dedicated to DFOS in geoengineering monitoring for many years. As a result, Nanjing University stands as a representative institution in this field in China. Out of the 20 authors, 11 are Chinese, which is attributable to the field’s close integration with engineering practice. The substantial number of engineering projects and high research efficiency in China contribute to the relatively abundant research achievements by Chinese scholars, who are gradually gaining international recognition for their research capabilities. While Chinese scholars lead in publication quantity in this field, their research primarily focuses on deformation and temperature monitoring in geoengineering. There is a relatively small proportion dedicated to the development and monitoring of emerging technologies. Moreover, Western scholars began their research earlier, which contributes to the relatively lower average citation count of Chinese scholars. In contrast, the scholars from the United States and Canada, despite having a smaller number of publications, command a significantly higher average number of citations. Their research spans various fields, including deformation monitoring in geoengineering, the development of fiber-optic sensors, geological carbon storage, and geothermal energy production, establishing them unequivocally as industry leaders.

In terms of both number of articles published and citations, Soga K. emerges as a prominent scholar in this field. Despite a relatively smaller number of recent articles, his recognition remains consistently high in this field. Yin J.H., on the other hand, is recognized as a highly respected scholar within the field in China. Notably, Zhu H.H. and Shi B. from China have sustained their research efforts in this field. While their impact may be limited, they have nonetheless made substantial contributions to the application of DFOS in geoengineering monitoring.

### 4.7. The Impacts of Publication Sources

Articles related to DFOS technology in geoengineering monitoring have been published in more than 1600 different journals. The number of journal sources publishing articles each year has increased from two in 1989 to 177 in 2023. We also examined the frequency distribution of the main sources of published papers in this field. The results indicate that conference proceedings have the highest number of publications, contributing a total of 585 articles, which account for 14.7% of the total publications. The top 20 journals collectively published 1269 articles, representing 32.0% of the total published papers. 

Table 6 lists the top 20 journals in terms of total publications. Among them, “Proceedings of SPIE” consists of conference journals and is the most productive journal for this field. However, the journal has an average citation count below five. This suggests that many scholars in this field prefer to share their research progress at academic conferences, but the quality of the papers may vary, leading to lower recognition. Apart from conference papers, journal articles remain a more widely accepted means of sharing research achievements for many scholars.

**Figure 13:**
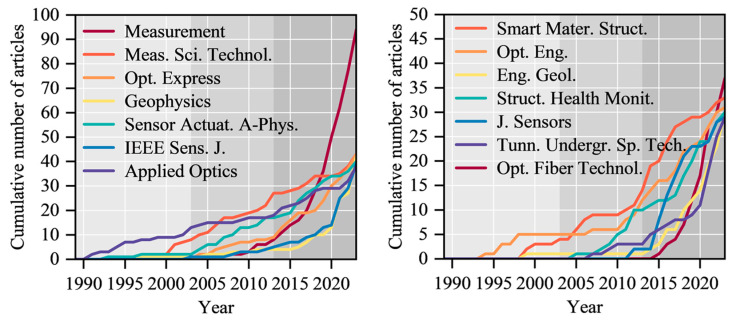
Top 14 publication sources with the highest number of journal publications over time.

Due to the significantly higher number of publications in conference proceedings compared to other academic journals, we have listed in Figure 13 the publication trends over time for the top 14 journals excluding conference proceedings. As shown in Figure 13, “Measurement” has seen significant growth in the number of publications in recent years. The growth rates of the remaining journals are relatively stable. The average citation counts to some extent reflect the quality of articles published in journals. From this perspective, “Engineering Structures” (77.81), “Geophysical Research Letters” (44.6), “Sensors and Actuators A-Physical” (32.6), “Scientific Reports” (32.5), and “Optics Express” (30.16) are the top five high-quality journals in this field, with articles that are more valuable in terms of referencing. These core journals collectively play a crucial role in the research of distributed monitoring in geotechnical engineering.

### 4.8. Keywords and Research Trends

**Figure 14:**
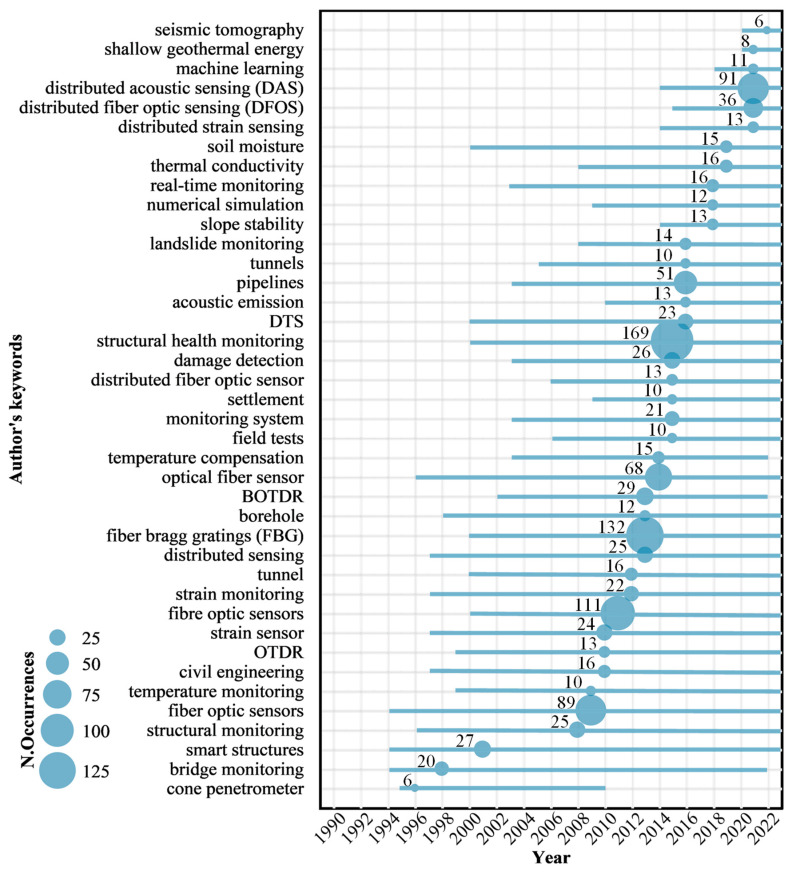
Temporal trends of keyword development. The x-axis represents the year, and the y-axis represents the author’s keywords. The blue lines indicate the time elapsed between the first quartile and the third quartile of a publication, while the dots indicate the median year of the publication and their size reflects the number of publications.

In this paper, we collected keywords from 3970 articles related to DFOS technology in geoengineering monitoring, involving more than 10,000 authors. These keywords were analyzed to identify research trends. Figure 14 illustrates the temporal development trends of keywords in articles from 1989 to 2023. For each keyword, the blue horizontal line represents the evolutionary process, with the blue dots corresponding to the median time when the keyword appeared, and the dot size reflects the frequency of the respective keyword. From the figure, it can be observed that before 2003, various sensing technologies, such as “DTS”, “OTDR”, “FBG”, and “BOTDR”, and many application scenarios, such as “civil engineering”, “bridge monitoring”, “tunnel”, “borehole”, had already appeared but had not yet become hotspots in research. As time progressed, research hotspots continuously evolved with new research directions emerging, reflecting the potential application of DFOS in geotechnical monitoring. Once a research hotspot represented by a keyword emerges in this field, scholars tend to continue studying it, as shown in Figure 14, in which different keywords appear at different times, most of which persist from their inception to the present day. This trend also contributes to the increasing diversity in the research areas discussed earlier.

The median time when the keyword appeared represents the widespread research focus on that particular research direction. In this context, before 2003, keywords like “cone penetrometer”, “bridge monitoring”, and “smart structures” appeared, indicating the earliest applications of DFOS technology in engineering-related structural monitoring fields. This corresponds to the increasing number of publications in engineering and instrumentation fields as shown in Figure 5. Subsequently, from 2004 to 2013, keywords such as “fiber optic sensors”, “OTDR”, “fiber Bragg gratings (FBG)”, “BOTDR”, “temperature monitoring”, and “civil engineering” emerged. During this period, a diverse range of fiber-optic sensing technologies were applied for on-site or laboratory monitoring, emphasizing multi-parameter and multi-scenario monitoring. From 2014 to the present, research hotspots have continued to emerge, and there have been more frequent shifts in research directions. The appearance of keywords such as “settlement”, “damage detection”, “pipelines”, and “soil moisture” indicates the expanding application scenarios for DFOS technology. During this phase, DFOS technology has transitioned from single-point, single-line monitoring to system monitoring, making advances in real-time monitoring capabilities. Moreover, scholars have increasingly explored interdisciplinary approaches, integrating technologies like numerical simulation with fiber-optic monitoring, which has gradually become a research hotspot. In recent years, the appearance of keywords such as “machine learning”, “seismic tomography”, and “distributed acoustic sensing (DAS)” indicates a growing trend towards technological innovation and interdisciplinary integration in the field. The introduction of new sensing technologies has broadened the parameters for distributed monitoring, and the abundance of monitoring data has spurred scholars to analyze and address problems from the perspectives of big data and machine learning.

Overall, the right side of Figure 13 has more and larger blue dots, indicating that in the past decade, there have been numerous newly added research directions in the field, and a substantial number of related articles have been published. This reflects the research trends [87]. In terms of monitoring technology, FBG is the most used monitoring method. In recent years, the emergence of new technologies such as weak fiber Bragg grating and ultra-weak fiber Bragg grating has not only retained the high precision advantages of FBG monitoring but also enabled near-distributed monitoring. This overcomes the limitations of traditional FBG monitoring in terms of distance and has been increasingly applied by scholars in on-site monitoring, yielding significant results [13,95]. Regarding data processing, machine learning and deep learning have emerged as new technological means in recent years. Leveraging diverse on-site monitoring data, these methods are employed for pattern recognition, data mining, and trend prediction [96,97]. Furthermore, the application of DFOS technology is expected to expand in the future. For example, with advancements in computer technology, it has become possible to filter out noisy data and identify valuable information using DAS technology. The effectiveness of fiber-optic sensing technology has been gradually confirmed in monitoring infrastructure health, identifying seismic and volcanic events, environmental monitoring, and other fields. In the future, this high-sensitivity, high-precision monitoring technology is poised to integrate with the Internet of Things (IoT), offering opportunities for real-time monitoring, forecasting, and early warning systems for relevant information. Moreover, within sensor development, durability and stability have long been concerns for scholars both within and outside the field. The challenge lies in ensuring the accuracy of monitoring results while achieving long-term monitoring, balancing effectiveness with cost efficiency. However, the industry currently lacks unified international standards and guidelines. Efforts from practitioners and distinguished scholars worldwide are needed to apply the latest fiber-optic sensing technologies effectively in engineering design, construction, and monitoring standards, ensuring the technology’s efficacy and safety.

## 5. Discussion

Currently, numerous review articles on DFOS have been published in this field, providing significant insights into the research history and current status of DFOS. Our search results identified 62 review articles, accounting for only 1.56% of the total publications. This indicates that the field is highly practical, with a primary focus on original research. It also suggests that although many new sensing technologies have been proposed and applied, a substantial proportion of research still utilizes relatively mature DFOS technology, and the existing review articles adequately address the overall research progress. These reviews cover the development of fiber-optic sensing technology, comparisons of different types of fiber-optic sensing technologies, and their applications in geotechnical and geological engineering monitoring. They offer valuable references for understanding the perspectives and research priorities of different research teams and scholars regarding DFOS technology.

Generally, the number of citations a paper receives reflects its impact; the greater the impact, the more people the information provided in an article is disseminated to. From the year of publication to the present, the top five most cited articles are by Lu et al. [98], Joe et al. [99], Li et al. [7], He et al. [100], and Hong et al. [101]. Interestingly, when considering the country of review articles, the results align with the above analysis of publication numbers by country, with review articles from the United States, China, and South Korea standing out. The most cited article is by an American scholar, detailing the principles and applications of various DFOS technologies. Despite being published relatively recently, it ranks first in total and average citations due to the high impact factor of the journal and the quality of the article, making it an exemplary review in this field. The second most cited article is a review by South Korean scholars on the use of fiber-optic sensors for environmental monitoring, covering applications in petroleum engineering, civil engineering, and agricultural engineering, indicating their significant influence. Notably, three of the top five most cited articles are from China, focusing on the application of DFOS technology in civil engineering, bridge engineering, and geotechnical engineering. This indicates that, overall, Chinese scholars have an advantage in the number of publications in this field, and some of them produce high-quality research. Their work helps researchers understand key issues and advancements in the field, thereby promoting knowledge dissemination.

In contrast with the aforementioned review articles, this paper aims to conduct a bibliometric analysis of the application of DFOS technology in geoengineering monitoring. Our goal is to help practitioners understand the historical and current developments of DFOS technology, better identify high-level research countries, institutions, and individuals in the field, and more effectively track academic trends. Bibliometrics can effectively interpret and describe a large number of publications [102]. Having a solid understanding of bibliometric principles is essential for conducting analyses. Although this study has made efforts to analyze and distill the content as comprehensively as possible, it has certain limitations.

Firstly, this research is based on a portion of the content from the WoS core database. However, the WoS database does not cover all published outcomes, resulting in potential unfairness to works published in other languages (such as Chinese, Russian, Japanese, etc.). Additionally, the study applied filtering criteria related to geotechnical engineering distributed monitoring, which may have led to the omission or redundancy of studies in the literature relevant to this field.

Furthermore, there are limitations in the analysis of author impact and research trends. When assessing author impact, it was observed that geotechnical engineering fiber-optic monitoring was only a minor research direction for some authors. Authors with high impact in other research areas may have inflated H-index values, making it inappropriate to analyze them together with authors solely focused on geotechnical engineering fiber-optic monitoring. Regarding the analysis of research trends, we chose author keywords for our analysis, considering that authors know their own articles the best. However, due to the extraction and merging of words, there might be duplicates of synonymous terms. 

## 6. Conclusions

We conducted a comprehensive review of the application of DFOS technology in geoengineering monitoring, utilizing a scientometric analysis facilitated by the bibliometrix R-package. A dataset comprising 3970 relevant papers spanning from 1989 to 2023 was extracted from the WoS core repository. Our analysis provides valuable insights into the development, impact, and future trends of DFOS technology in the realm of geoengineering monitoring. The key conclusions drawn from our study are outlined below:

(1) Over the past 35 years, DFOS technology has played an important role in modern infrastructure monitoring and disaster prevention. Engineering has become the subject category with the largest number of articles published in this field. Due to its high-accuracy sensors, cost-effective instruments, and capability of taking multi-parameter measurements, FBG has an advantage out of the different technology categories.
(2) The United States, China, and the United Kingdom emerged as major contributors, with China surpassing the United States in total publications in 2016. However, challenges persist for Chinese publications in achieving a comparable level of citations or impact.
(3) Institutions such as the Nanjing University and Dalian University of Technology led in terms of publication count, but the United States Department of Energy and the University of California system showcased a superior impact with fewer publications. The journal analysis highlighted the productivity of conference journals, emphasizing the need for a balanced approach considering both impact and quantity.
(4) The development trend of distributed monitoring in geotechnical engineering shows a dynamic trajectory. Initially focused on using fiber optics for structural health monitoring, recent research indicates a more diverse environment with a shift towards interdisciplinary collaborations. Scholars in the field are increasingly integrating emerging technologies, like machine learning and distributed acoustic sensing. This suggests a future characterized by advanced monitoring technologies, a reliance on data-driven approaches, diverse application scenarios, and the development of sensors that combine durability and stability.

## Figures and Tables

**Table 1:** DFOS technologies and their features in geoengineering monitoring.

Sensing Type	Sensing Technology	Sensing Parameters	Sensing Accuracy	Maximum Sensing Distance (km)	Spatial Resolution (m)	Sampling Resolution (m)	Advantages	Limitations
Quasi-distributed	FBG [42]	Strain	±1 με	-	-	-	Cost-effective, high reliability, corrosion resistance, high sensitivity, easy to implement multiplexing	Possibility of missed detection, grating extinction phenomenon at high temperature
		temperature	±0.1 °C					
	UWFBG [13]	Strain	±1 με	-	-	-	High resolution, high sensitivity, multipoint-capable, broadband	High equipment cost, complex fabrication, limited dynamic range
		temperature	±0.1 °C					
Fully distributed	BOTDR [42]	Strain	±10 με	80	1.0	0.05	Long-range sensing, distributed sensing capability, non-destructive testing	Costly equipment, environmental sensitivity, limited resolution for very long distances
		temperature	±1.0 °C					
	BOFDA [43]	Strain	±2 με	80	0.2–2.5 *	0.05	Long-range sensing, high accuracy, high spatial resolution	Costly equipment, limited dynamic range, complex system setup
		temperature	±0.1 °C					
	BOTDA [44]	Strain	±20 με	25	0.05–1 *	0.01	High sensitivity, high spatial resolution, short testing time	High equipment cost, limited resolution for extremely long distances
		Temperature	±1.0 °C					
	ROTDR [42]	Temperature	±0.3 °C	16	0.5–3 *	0.05	Simple setup, cost-effective, long-range sensing capability	Signal degradation, limited dynamic range
	OFDR [45]	Strain	±1 με	0.07	0.00065–0.01 *	0.0003	High spatial resolution, high sensitivity, simultaneous multi-parameter sensing	High equipment cost, short measurement distance, long data acquisition time
		Temperature	±0.1 °C					
	OTDR [46]	Signal loss	-	260	-	0.04–40 **	Long-range measurement, high fault detection accuracy	High equipment cost, sensitivity to spurious reflections
	Φ-OTDR [47]	Vibration	-	40	1–10 *	0.01	Long-range capability, high sensitivity, enhanced detection of small disturbances	High equipment cost, complex data processing, environmental sensitivity

Note: * Varies with sensing distance; ** Varies with sensing distance and pulse width.

**Table 2:** Typical applications of DFOS technology in geoengineering monitoring.

Typical Scenarios	Sensing Parameters	Sensing Technology	Applications Sites	References
Landslides	Strain, Temperature, Soil moisture content, Displacement	FBG, UWFBG, BOTDR, BOTDA	Three Gorges Reservoir, China; Izumo landslide, Japan; Basilicata, Italy	[10,13,48,49,50]
Debris flows	Displacement, Stress, Vibration	FBG	Weijiagou, China; Nautou county, China	[51,52,53,54]
Ground subsidence and land fissures	Strain, Displacement	BOTDA, BOTDR	Wuxi, China; Ebro Valley, Spain	[55,56]
Tunnels	Displacement, Strain	BOTDA, OFDR, OTDR	Mass Rapid Transit (MRT) tunnel, Singapore; Ebersviller tunnel, French; Suzhou Metro Line 3, China; Heinenoordtunnel, Netherlands	[43,57,58,59]
Pipelines	Strain	FBG	Colombian pipeline, United States; Three Gorges Reservoir, China	[60,61]
Railways	Temperature, Strain, Displacement	FBG, ROTDR	Qinghai-Tibet Railway, China; Stagecoach Supertram tramway, United Kingdom; Santo Stefano Magra railway, Italy	[62,63,64,65]

**Table 3:** Characteristics of major technology categories from 1989 to 2018.

Technology	A	TC	H-Index	TC/A	1st Year
FBG	859	5712	39	12.31	1997 [82]
OTDR	111	926	16	11.02	1994 [83]
BOTDR	109	1963	21	19.44	2002 [84]
BOTDA	105	1280	20	15.06	2008 [85]

A: total number of articles; TC: number of citations for all articles; TC/A: number of citations per article; 1st Year: the first year of relevant article published.

**Table 4:** The top 20 institutions with the most publications in this field.

Institution	Country	TA	TC	TC/TA
Nanjing University	China	169	2910	17.22
Dalian University of Technology	China	107	2579	24.1
United States Department of Energy	United States	104	3165	30.43
Chinese Academy of Sciences	China	97	1103	11.37
Harbin Institute of Technology	China	86	1193	13.87
Swiss Federal Institutes of Technology Domain	Switzerland	85	2014	23.69
Hong Kong Polytech University	China	68	1976	29.06
University of California System	United States	68	1980	29.12
Centre National de la Recherche Scientifique	France	66	1132	17.15
University of Cambridge	United Kingdom	60	1990	33.17
Helmholtz Association	Germany	58	1398	24.1
Wuhan University of Technology	China	52	275	5.29
China University of Mining Technology	China	51	436	8.55
Lawrence Berkeley National Laboratory	United States	49	1629	33.24
Southeast University China	China	44	672	15.27
Helmholtz Center Potsdam GFZ German Research Center for Geosciences	Germany	42	838	19.95
Chang’an University	China	19	366	19.26
Naval Research Laboratory	United States	19	332	17.47
University of Birmingham	United Kingdom	19	454	23.89
University of Trento	Italy	19	339	17.84

TA: total number of articles; TC: number of citations for all articles; TC/TA: number of citations per article.

**Table 5:** The top 20 core authors ranked by H-index in this field.

Author	H-Index	TA	TC	TC/TA	Country	Affiliation
Zhu H.H.	28	84	2011	23.94	China	Nanjing University
Shi B.	26	120	2119	17.66	China	Nanjing University
Yin J.H.	19	37	1140	30.81	China	Hong Kong Polytechnic University
Glisic B.	18	57	1062	18	United States	Princeton University
Wei G.Q.	16	37	708	19.14	China	Suzhou Nanzee Sensing Technol Co., Ltd.
Zhang C.C.	16	36	692	19.22	China	Nanjing University
Ansari F.	15	29	795	27.41	United States	University of Illinois at Chicago
Soga K.	15	27	1262	46.74	United States	University of California, Berkeley
Inaudi D.	14	60	915	15.25	Switzerland	Smartec SA
Zhang L.	14	28	585	20.89	China	China University of Geosciences
Pei H.F.	14	21	681	32.43	China	Dalian University of Technology
Xu D.S.	13	16	489	30.56	China	Wuhan University of Technology
Zhang D.	12	30	479	15.97	China	Nanjing University
Benmokrane B.	12	17	743	43.71	Canada	University of Sherbrooke
Hong C.Y.	11	21	717	34.14	China	Shenzhen University
Chai J.	10	26	280	10.77	China	Xi’an University of Science and Technology
Grattan K.T.V.	10	20	352	17.6	United Kingdom	University of London
Schenato L.	10	19	319	16.79	Italy	National Research Council–Research Institute for Geo–Hydrological Protection
Reinsch T.	10	17	338	19.88	Germany	German Research Centre for Geosciences
Minardo A.	10	15	284	18.93	Italy	Università della Campania Luigi Vanvitelli

TA: number of total articles; TC: number of citations for all articles; TC/TA: number of citations per article.

**Table 6:** The top 15 publication sources ranked by the total number of articles in this field.

Publications	Type	TA	TC/TA	H-Index	IF
Proceedings of SPIE	C	585	3.48	18	--
Measurement	J	94	21.27	27	5.2
Measurement Science and Technology	J	43	26.21	19	2.7
Optics Express	J	43	30.16	16	3.2
Geophysics	J	42	10.6	13	3.0
Sensors and Actuators A-Physical	J	40	32.6	18	4.1
IEEE Sensors Journal	J	39	13.62	14	4.3
Applied Optics	J	38	17.37	13	1.7
Optical Fiber Technology	J	37	11.59	12	2.6
Smart Materials and Structures	J	33	29.12	19	3.7
Optical Engineering	J	31	6.32	8	1.1
Engineering Geology	J	30	23.33	15	7.6
Structural Health Monitoring-An International Journal	J	30	26.6	17	5.7
Journal of Sensors	J	29	11.31	10	1.4
Tunnelling and Underground Space Technology	J	29	29.66	18	6.7
Engineering Structures	J	27	77.81	17	5.6
Scientific Reports	J	26	32.5	13	3.8
Structural Control & Health Monitoring	J	26	27.35	14	3.8
Geophysical Research Letters	J	25	44.6	16	4.6
Journal of Geophysical Research Solid Earth	J	22	21.45	10	3.9

C: conference paper; J: journal; TA: total number of articles; TC/TA: number of citations per article; IF: Impact factor for 2022–2023.

## Data Availability

Data are contained within the article.
